# A pollution gradient contributes to the taxonomic, functional, and resistome diversity of microbial communities in marine sediments

**DOI:** 10.1186/s40168-019-0714-6

**Published:** 2019-07-15

**Authors:** Jiarui Chen, Shelby E. McIlroy, Anand Archana, David M. Baker, Gianni Panagiotou

**Affiliations:** 1 Leibniz Institute for Natural Product Research and Infection Biology, Hans Knoll Institute, Beutenbergstrasse 11a, Jena, 07745 Germany; 2 Swire Institute of Marine Science, The University of Hong Kong, Hong Kong SAR, China; 3 School of Biological Sciences, Faculty of Science, The University of Hong Kong, Kadoorie Biological Sciences Building, Pok Fu Lam Road, Hong Kong SAR, China; 4 Department of Microbiology Li Ka Shing Faculty of Medicine, The University of Hong Kong, Hong Kong SAR, China; 5 Systems Biology & Bioinformatics Group, School of Biological Sciences, Faculty of Science, The University of Hong Kong, Hong Kong SAR, China

**Keywords:** Metagenomics, Pollution concentration, Marine sediments, Antibiotic resistance genes

## Abstract

**Background:**

Coastal marine environments are one of the most productive ecosystems on Earth. However, anthropogenic impacts exert significant pressure on coastal marine biodiversity, contributing to functional shifts in microbial communities and human health risk factors. However, relatively little is known about the impact of eutrophication—human-derived nutrient pollution—on the marine microbial biosphere.

**Results:**

Here, we tested the hypothesis that benthic microbial diversity and function varies along a pollution gradient, with a focus on human pathogens and antibiotic resistance genes. Comprehensive metagenomic analysis including taxonomic investigation, functional detection, and ARG annotation revealed that zinc, lead, total volatile solids, and ammonia nitrogen were correlated with microbial diversity and function. We propose several microbes, including *Planctomycetes* and sulfate-reducing microbes as candidates to reflect pollution concentration. Annotation of antibiotic resistance genes showed that the highest abundance of efflux pumps was found at the most polluted site, corroborating the relationship between pollution and human health risk factors. This result suggests that sediments at polluted sites harbor microbes with a higher capacity to reduce intracellular levels of antibiotics, heavy metals, or other environmental contaminants.

**Conclusions:**

Our findings suggest a correlation between pollution and the marine sediment microbiome and provide insight into the role of high-turnover microbial communities as well as potential pathogenic organisms as real-time indicators of water quality, with implications for human health and demonstrate the inner functional shifts contributed by the microcommunities.

**Electronic supplementary material:**

The online version of this article (10.1186/s40168-019-0714-6) contains supplementary material, which is available to authorized users.

## Background

Over the last two centuries, human activities such as coastal development and nutrient discharge from wastewater have driven major changes in marine biodiversity [1, 2]. Nutrient pollution, or eutrophication, detrimentally affects global marine ecosystems by impacting the diversity and function of a wide range of foundational species such as seagrass, oysters, corals, and other metazoans, and microbes including bacteria and viruses [3–5]. Eutrophication also negatively impacts marine coastal sediments that harbor microbial communities in high abundance and diversity. Not only are marine sediments hot spots for nitrogen and carbon cycling [6], they also serve as a long-term reservoir of terrigenous and aquatic pollutants [7]. Therefore, understanding the mechanisms by which water pollution affects the diversity and function of microbial populations in sediments is paramount.

Until recently, researchers used environmental microbes and their genetic markers as indicators for pollution [8, 9]. These studies began to reveal that microorganisms living inside marine sediments correlated well with pollutants such as trace metals and persistent organic compounds [10] and likely impacted sedimentary niche changes [11]. However, this methodology has not shed light on the overall genetic profiles of the communities.

Advances in high-throughput sequencing have revolutionized the detection of genes in complex environmental communities, offering a more promising avenue for comprehensive genetic profiling [12]. Through shotgun metagenomic and metabarcoding analyses, studies have revealed that sediments in urbanized areas with higher pollution levels may be enriched in human pathogens [13], such as those that cause ciguatera poisoning, avian influenza [14], and gastroenteritis [15]. In addition to changes in taxonomic profiles, changes in gene abundances have also been observed in impacted microbial communities, including genes associated with metabolic processes such as denitrification [16] and genes related to virulence/defense and stress response such as antibiotic resistance genes (ARGs) [17]. Although the aforementioned studies demonstrate that eutrophication alters microbial communities and genetic composition, very few comprehensive studies combining all taxonomic, functional, and resistance profiles have been performed to examine the influence of a well-constrained pollution gradient on the metagenomic profiles of microbial communities and the possible pathogenic risks for both humans and the environment. Moreover, a significant knowledge gap remains in understanding the associated impacts of pollution on influencing microbiome communities.

Furthermore, Hong Kong, which is located within the fastest developing area in southern China (the Greater Bay Area) has a population of approximately 7.5 million, and is situated at the mouth of the Pearl River Delta (PRD) offering one of the greatest opportunities to investigate the influence of pollution on the marine sediment microbiome. The PRD is also regarded as the Pearl River Estuary (PRE), through which the Pearl River enters the South China Sea. During the 1980s to the 1990s, Hong Kong was rapidly developed and subjected to land reclamation and population migration. The pollution footprint increased with a lagging investment in wastewater treatment. Therefore, eutrophication has contributed to losses of foundational species such as hard corals in many areas [18, 19]. Tolo Harbour [20] is a typical area suffering from eutrophication and is an enclosed bay located in northeast Hong Kong. Tolo Harbour was previously coral-dominated but the coral was gradually replaced by other organisms including algae and suspension feeders due to rapid coastal development inshore. However, coral communities still exist in some offshore regions of Tolo Harbour.

**Fig. 1:**
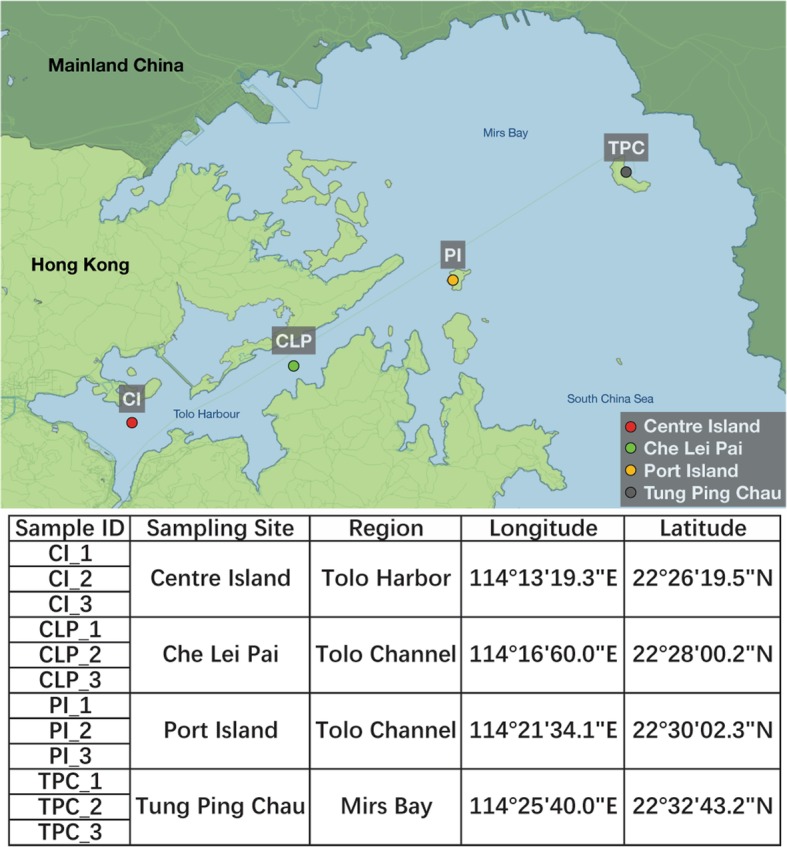
Sampling information. A map of Tolo Channel (including Tolo Harbour) and Mirs Bay shows the four sampling sites marked in circles of different colors: Centre Island, Che Lei Pai, Port Island, and Tung Ping Chau. Samples were collected from the sediments at 6–10-cm depth. The table shows the information of each sample including sampling site, region, longitude, and latitude

Thus, in our study, we examined four field sites in Tolo Harbour that have different degrees of eutrophication, including Centre Island (dead oyster reef), Che Lei Pai (sandy bottom with sparse vegetation), Port Island (50% hard coral cover), and Tung Ping Chau (75% coral cover) (Fig. 1). These sites were selected based on seven water quality gradients (Additional file 1: Table S1) and their coral species richness, which made them ideal regions to study the impact of pollution. By using a shotgun metagenomic approach, we assessed the microbial community composition, the functional characteristics of the microbial community, the prevalence of pathogenic bacteria, and the abundance and dissemination potential of ARGs in marine sediments. Lastly, we evaluated the differences in microbial communities among sampling sites in connection to representative pollution parameters. Our study revealed that the antibiotic resistome composition of marine sediment microbial communities is significantly different depending on the pollution concentration and, moreover, the distribution of microbial communities is highly associated with the pollution parameters.

## Results

### Sequencing results

The 12 sediment samples subjected to metagenomic sequencing generated 138.6 Gb of raw data (average of 11.55 Gb per sample). Quality filtering reduced the average raw data to 6.66 Gb of filtered sequencing data per sample. On average, 85.3% of the mapped reads were classified as bacteria per sample, followed by archaea at 12.1% and eukaryotes at 2.6%. The percentage of bacterial reads was significantly higher in samples from Centre Island than those from Tung Ping Chau (*P* = 0.0172, Student’s *t* test) whereas the percentage of archaeal and eukaryotic reads were significantly lower (*P* = 0.0222 and *P* = 0.0317, respectively). Moreover, the percentage of bacterial reads was also significantly higher in samples from Centre Island compared to those from Port Island (*P* = 0.0199).

### Pollutant concentration profiles

The normalized concentrations of seven geochemical indicators were used to represent environmental pollution levels at each sampling site (Additional file 1: Table S1). Zinc (Zn), lead (Pb), and copper (Cu) are common heavy metal pollutants of marine environments with arsenic regarded as highly toxic. Organic pollution was represented by ammonia nitrogen (NH_3_-N), chemical oxygen demand (COD) and total volatile solids (TVS). Centre Island, the innermost site within Tolo Harbour, had the highest concentrations of all pollutants with a general trend of decreasing pollutant concentrations with distance from the inner harbor (Fig. 1). Port Island and Tung Ping Chau exhibited similar levels of Zn, Pb, Cu, arsenic, and NH_3_-N.

### Comparative analysis of microbial communities in a pollution gradient

**Fig. 2:**
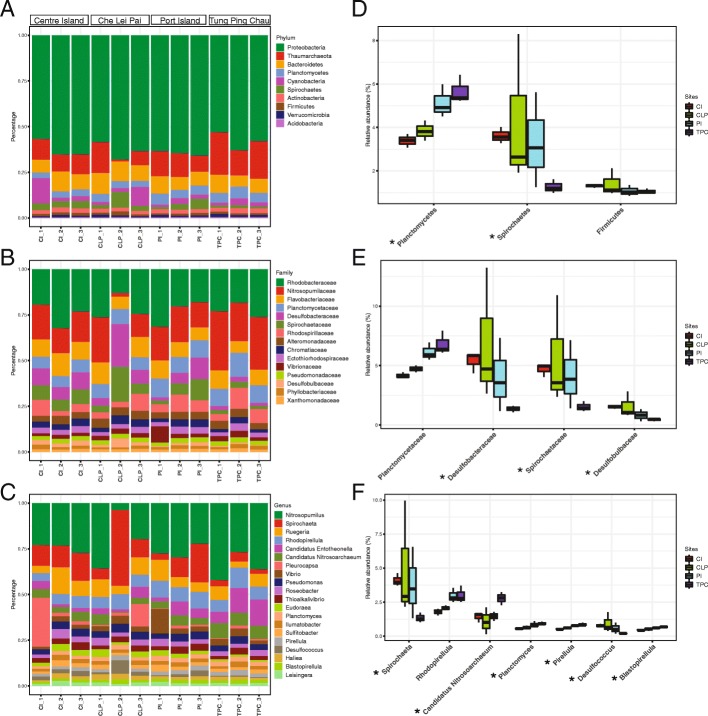
Comparative analysis of the prokaryotic communities among the sampling sites. **a**, **b**, **c** Relative abundance of the most abundant microbes across sites at the phylum, family, and species levels, respectively. **d**, **e**, **f** Relative abundance of the most abundant microbes which have significantly different abundances among sites. Microbes with asterisk retain strong differentiation after FDR correction (adjust *q* value < 0.1)

We constructed the total prokaryotic profile by extracting all bacterial and archaeal reads among samples at different taxonomic levels from phylum to genus. The most abundant prokaryotic phyla were *Proteobacteria* (60.3 ± 4.3%, SD), *Thaumarchaeota* (12.2 ± 5.7%), and *Bacteroidetes* (8.46 ± 1.7%) (Fig. 2a). The five most abundant prokaryotic families were *Rhodobacteraceae*, *Nitrosopumilaceae*, *Flavobacteriaceae*, *Planctomycetaceae*, and *Desulfobacteraceae*, which comprised on average ~ 42% of the prokaryotic communities (Fig. 2b). At the genus level, *Nitrosopumilus* (9.1 ± 3.8%) was the most abundant across all communities (Fig. 2c). The comparisons on the relative abundance of prokaryotes among the four sampling sites at the above described taxonomic levels (Additional file 2: Table S2 and Additional file 4: Table S4) revealed that Centre Island and Tung Ping Chau had the most dissimilar microbial communities with the largest number of significantly different prokaryotes (at every taxonomic level). Notably, *Planctomycetes* was the only phylum showing a significant difference among the four sampling sites (*P* = 0.030, Kruskal-Wallis test), with an increasing abundance from Centre Island to Tung Ping Chau (Fig. 2d). The abundance of *Planctomycetes* was significantly different in the comparisons between Centre Island and Tung Ping Chau (*P* = 0.014, Student’s *t* test), Centre Island and Port Island (*P* = 0.043), and Che Lei Pai and Tung Ping Chau (*P* = 0.020). Further investigation of its major family (*Planctomycetaceae*) and genus (*Planctomyces*) revealed the same tendency when comparing Centre Island to Tung Ping Chau (*P* = 0.022, Kruskal-Wallis test and *P* = 0.008, Student’s *t* test, respectively) (Fig. 2e). At the phylum level, we further observed that several human-related prokaryotes had significantly increased abundances in Centre Island compared to Tung Ping Chau, including *Spirochaetes*, a phylum that includes potential pathogens (*P* = 0.001, Student’s *t* test), and *Firmicutes*, one of the most abundant phyla in the human gut microbiome (*P* = 0.044) (Fig. 2f).

**Fig. 3:**
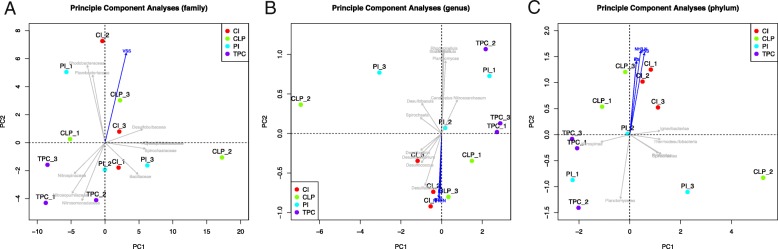
Community dissimilarities among sampling sites with pollutant vectors. **a**, **b**, **c** Principal component analyses for the microbes among sites at phylum, family, and species levels, respectively. Vector matrices of the significant pollution parameters are shown by individual black lines with an arrow. The significant microbes are shown in gray

To evaluate the community diversity of each site and the differences in microbial composition among them, we calculated both the alpha and beta diversity using different indices. For the alpha diversity, neither the Shannon nor Simpson diversity indices showed any significant difference among sampling sites (Additional file 7: Figure S2). Notably, the principle component analyses (PCA) based on the most abundant prokaryotes revealed significantly different communities among the four sites at the phylum level (*P* = 0.048, PERMANOVA). Furthermore, we observed clear separations of the microbial communities between Centre Island and Tung Ping Chau at phylum and genus level (*P* = 0.047 and *P* = 0.049, respectively) (Fig. 3). Four pollution parameters (zinc, lead, total volatile solid, and ammonia nitrogen) were significantly correlated with communities at either phylum or genus levels (*P* < 0.05, Additional file 5: Table S5) (Fig. 3). At the phylum level, the pollution parameters can further serve as potential negative indicators of the distribution of *Thaumarchaeota* and *Planctomycetes,* which were significantly enriched in Tung Ping Chau (*P* = 0.008, Student’s *t* test). At the genus level, the distribution of *Desulfatitalea*, *Desulfococcus*, *Desulfobacterium*, and *Desulfovibrio* are positively correlated with the representative parameters to different extents, while four genera of *Plactomycetes* (*Planctomyces*, *Blastopirellula*, *Pirellula*, and *Rhodopirellula*) were negatively correlated. At the family level, total volatile solids was the best indicator of the community distribution, and interestingly, was negatively correlated with three nitrifying bacteria (*Nitrosomonadaceae*, *Nitrosopumilaceae*, and *Nitrospinaceae*).

In addition to the abundance of microbes in the sediment, we also investigated the replication activity of different species, which could reflect changes in active communities. Therefore, we selected the 30 most .abundant species among all samples and applied the iRep [21] algorithm for evaluating the replication rate. Among these species, the replication rates of 14 species were estimated based on sufficient genome coverage among all the samples. *Ruegeria conchae*, which belongs to *Rhodobacteraceae*, the most abundant family in all samples, had on average the highest replication rate compared to other species across all sites (*P* < 10^−8^, Student’s *t* test) (Additional file 8: Figure S3). By comparing the replication rate among different sampling sites, we observed that *Candidatus Nitrosopumilus* sp. *NF5* had higher replication rates in Port Island and Tung Ping Chau than in Centre Island and Che Lei Pai (*P* = 0.027). *Candidatus Nitrosoarchaeum koreensis* also showed a higher replication rate in Tung Ping Chau than in Centre Island (*P* = 0.031).

### Functional shifts contributed by microcommunities

**Fig. 4:**
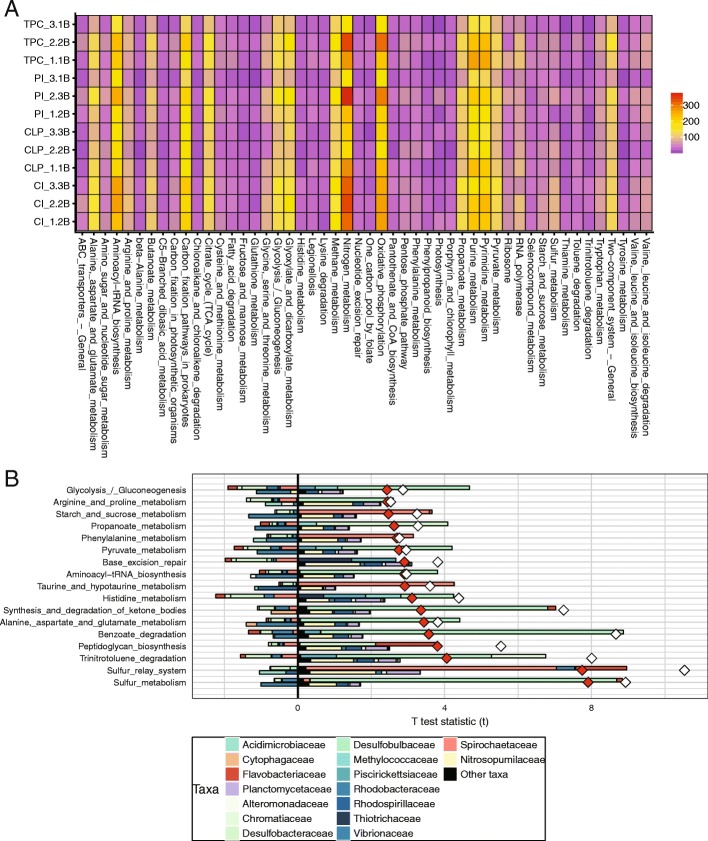
KEGG Orthology annotation and taxonomic drivers of functional shifts between sites. **a** Heat map of the top 50 abundant KEGG pathway annotations. **b** Taxon-level shift contribution profiles for significantly different KO pathways between Centre Island (case group) and Tung Ping Chau (control group) based on the top 25 abundant families. Taxonomic contributors to functional pathways differences between Centre Island (case group) and Tung Ping Chau (control group) based on the top 25 abundant families. The white and red diamonds refer to the taxa-based and functional-based shift scores, respectively

We annotated our metagenomics sequencing data using the KEGG database and constructed the pathway profiles for the microbial communities of each sampling site. “Nitrogen metabolism” was the most abundant functional pathway among all samples (6.1%), followed by “oxidative phosphorylation” (5.2%) and “aminoacyl-tRNA biosynthesis” (4.8%) (Fig. 4a). We implemented FishTaco [22], a novel algorithm for integrating taxonomic and functional comparative analyses to accurately quantify family-level contributions to functional shifts. We performed the analyses by using the microbial communities of Centre Island and Tung Ping Chau, which were the most dissimilar in terms of pollution concentration and were found to have the most diverse prokaryotic composition according to the above analyses.

After comparing the annotated KEGG pathways between Centre Island and Tung Ping Chau, 17 pathways showed significant enrichment at Centre Island (Additional file 3: Table S3). We further extracted the 25 most abundant families in these two sites that comprised over 72% of all the microbial communities. The Pearson correlation coefficient was calculated based on the pathway profile and the abundant family profile (Additional file 9: Figure S4). The results showed an average Pearson correlation coefficient of 0.91, suggesting a tight correlation between taxon and functional profiles. *Desulfobulbaceae* was found to be the main driver of the enrichment of the sulfur metabolism and benzoate degradation pathways in Centre Island. Moreover, *Spirochaetaceae* served as the driver of the sulfur relay system (also known as sulfur transfer system) in Centre Island. In addition, the trinitrotoluene degradation pathway was found to be enriched in Centre Island and was driven by *Chromatiaceae* and *Nitrosopumilaceae*. (Fig. 4b). To further confirm our findings, Spearman correlation between the above pathways and families among the four sites were showing overall a relatively good consistency with the Pearson correlation (except the correlation between Spirochaetaceae and sulfur relay system) (Additional file 10: Figure S5).

### Putative pathogens and antibiotic resistome

**Fig. 5:**
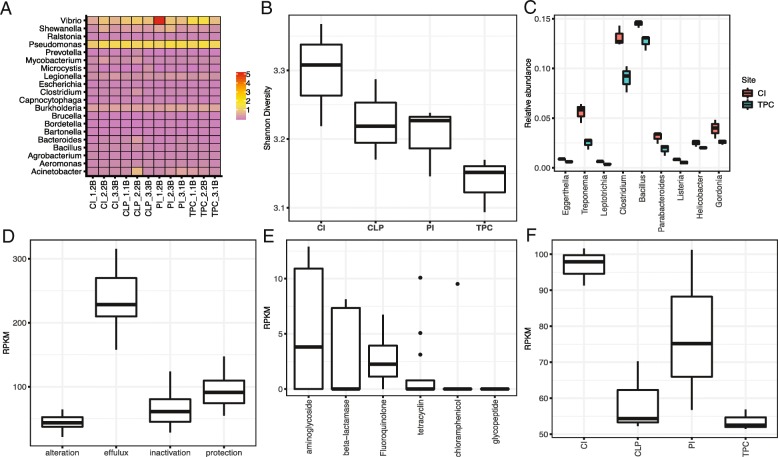
Comparisons of abundances of potential pathogens and antibiotic resistant genes (ARGs) among samples. **a** Heat map based on relative abundances of potential pathogenic genera in different samples. **b** Comparisons of pathogenic alpha diversity among sampling sites. **c** Comparisons of significantly different pathogenic genera between Centre Island and Tung Ping Chau. **d** Total relative abundance of each ARG mechanism among the sampling sites. **e** Total ARG profile of the samples. **f** Comparisons of total ARG abundances from each site

By aligning the taxonomic profiles to a list of potential human pathogens compiled from three different sources [23–25], we discovered opportunistic pathogenic species from 148 genera that comprised 4.92–7.65% of all the microbial communities, among which *Vibrio* (1.27 ± 1.18%) was the most abundant (Fig. 5a). Typical opportunistic pathogenic species of *Vibrio* including *V. cholera*, *V. parahaemolyticus*, and *V. vulnificus* were found at all the sampling sites. The potential opportunistic pathogenic species *P. aeruginosa* was found to have the second highest abundance (1.23 ± 0.10%). We further calculated the alpha diversity of the putative pathogenic communities among sampling sites using both the Shannon and Simpson indices (Fig. 5b). Alpha diversity decreased from Centre Island to Tung Ping Chau significantly, with nine putative pathogenic genera that were significantly more abundant in Centre Island compared to Tung Ping Chau (*P* < 0.05, Student’s *t* test) (Fig. 5c). Among them, *Bacillus* (0.14 ± 0.03%) was the most abundant putative pathogen (*P* = 0.037). *Clostridium*, another putative pathogen, was enriched in Centre Island compared to Tung Ping Chau (*P* = 0.015), mainly due to increased levels of the species *C. tetani*. In addition, two putative pathogenic species of *Treponema* (*T. denticola* and *T. pallidum*) were found in significantly higher abundances in Centre Island compared to Tung Ping Chau (*P* = 0.015).

Subsequently, we investigated the resistome profiles in the different sampling sites. Among ARG mechanisms, antibiotic efflux pumps (237.24 ± 47.7 RPM—reads per million reads) were the predominant resistance mechanism in the sediment communities, followed by target protection (95.74 ± 29.2 RPM), inactivation (65.29 ± 28.6 RPM) and target alteration (43.52 ± 13.2 RPM) (Fig. 5d). Annotation using ResFams identified 17 ARG families. Among the ARG families, five genes that have activities against specific antibiotics were detected including aminoglycoside, beta-lactamase, fluoroquinolone, tetracycline, and the chloramphenicol resistance gene (Fig. 5e), and there were no significant differences found among the sites. To evaluate the differences in the ARGs among the sediment samples, we compared the total abundances of the ARGs. The results showed that Tung Ping Chau had the lowest abundance of total ARGs, whereas Centre Island had the highest (*P* = 0.0001, Fig. 5). Further comparisons of the ARG families showed that three efflux pump genes, RND, baeR, and ABC, were significantly enriched in Centre Island compared to the other sites (*P* = 0.014, 0.047, and 0.033, respectively).

## Discussion

Microbial communities inhabiting marine sediments are extremely diverse due to the complex physicochemical gradients therein [26]. Community dynamics are affected by human activities, particularly those related to water chemistry and quality [13]. Hong Kong has a diverse distribution of highly urbanized and relatively undisturbed coastal environments [27] and provides a perfect opportunity to assess the relationship between microbial communities and different levels of perturbation. Earlier studies have investigated the community of bacteria and ARGs in marine sediments of Hong Kong using both clone libraries and metagenomics [7, 28, 29]. However, they were unable to identify clear patterns in either bacterial communities or ARGs. To fill this gap, we constructed a comprehensive pipeline for the proof-of-concept analysis of the microbiome communities, their functional diversity, and the resistome as affected by human activities and pollution levels. Throughout our analysis, we provided evidence of the impact of different pollution levels on the diversity of microbial communities and their functional-associated genes as well as the risks of increased levels of ARGs and pathogens.

Regarding the prokaryotic profile, we observed an enrichment of a number of phyla tightly associated with the human gut and terrestrial biomes in the most polluted areas. These phyla include *Spirochaetes*, a potential pathogenic phylum causing a wide range of diseases including leptospirosis, Lyme disease, and Alzheimer’s disease [30–32], and *Firmicutes*, which make up the largest portion of the human gut microbiome [33]. While the majority of sewage generated from the local population is treated and then redirected for discharge elsewhere (https://www.epd.gov.hk/epd/wqo_review/en/watQua.htm), the presence of *Firmicutes* may indicate that inputs remain and *Planctomycetes*, a widely distributed phylum found in a variety of marine environments [34, 35], exhibits a dose-response sensitivity effect to heavy metals [36] while generally resistant to high concentrations of inorganic nitrogen compounds. In our study, *Planctomycetes* was the fourth most abundant phylum among the samples, and its abundance was significantly decreased at Centre Island compared to Tung Ping Chau. Combined with the pollution data from the sampling sites, our study suggests that *Planctomycetes* may also serve as an important potential water quality indicator.

By evaluating the microbial diversity at the community level, we observed clear differentiation among the sampling sites—especially between Centre Island and Tung Ping Chau—and coherence with the distribution of representative pollutants. Interestingly, at the genus level, the four sulfate-reducing microbes (SRM) *Desulfatitalea*, *Desulfococcus*, *Desulfobacterium*, and *Desulfovibrio* were found to be positively correlated with pollution levels, suggesting that eutrophication increases the abundance of SRM. Previous studies in marine sediments of Hong Kong had also reported the relationship between SRM and pollution levels. For instance, pollutants may influence the relative abundance of SRMs to total microbes under a unimodal paradigm while low concentrations of pollutants will decrease relative abundance of SRMs in total microbes [7]. Moreover, with the clone library methods, unique SRM members related to the polluted harbor environment and estimated SRM richness correlated with several environment factors [29].

In our study, we also implemented a novel algorithm, based on sequencing coverage, which was used to calculate an index of replication (iRep) for estimating changes in the active microbial communities [21]. *Ruegeria conchae* had the highest replication rate among all sites, which is consistent with previous studies [37, 38]. These studies have shown that *Ruegeria* is present in a wide range of marine habitats, from coastal regions to deep-sea sediments, and constitute up to 25% of the total bacterial community. Interestingly, the active community analysis revealed additional changes in bacterial spp. replication rates along the water quality gradient. Two species of *Thaumarchaeota—Candidatus Nitrosopumilus sp. NF5* and *Candidatus Nitrosoarchaeum koreensis*—which are responsible for the major ammonia-oxidizing process [39, 40] in sediment, replicated faster in sites with high water quality (i.e., low ammonia; Additional file 1: Table S1). The concordance of replication rate with relative abundance not only confirms the adaptation of *Thaumarchaeota* to low-ammonia conditions but also reveals the mechanism by which it can maintain a high abundance.

When investigating pathogenicity among all sites, we observed that *Vibrio*, including its three opportunistic pathogenic species, *V. cholera*, *V. parahaemolyticus*, and *V. vulnificus*, were the most abundant. These disease-causing strains of *Vibrio* are associated with infectious diarrhea and are commonly carried by marine animals including crustaceans [41]. *Pseudomonas aeruginosa*, the second most abundant potential pathogenic species in our analysis, is a multidrug resistant pathogen and causes a wide variety of serious infectious diseases including meningitis, pneumonia, and septicemia [42–44]. The alpha diversity calculated from only putative pathogenic microbes decreased significantly from Centre Island to Tung Ping Chau, indicating that pollution inputs influence the local potential pathogen composition. Furthermore, nine opportunistic pathogenic genera were enriched at the most polluted site. Considering that coastal waters are the most impacted by human activities including recreation (e.g., swimming) and food supply (e.g., fishing and aquaculture), surface or water column sampling to assess public safety may overlook potential pathogen reservoirs in the sediment.

Based on the evidence above, we observed a connection between the extent of pollution and the abundance and diversity of pathogenic organisms in marine sediments. Pinpointing the drivers of these patterns is complex as feedback loops between both biotic and abiotic process function across multiple spatial and temporal scales. For example, healthy and biodiverse marine ecosystems buffer against the proliferation of bacterial pathogens [5, 45] but foundational species can be sensitive to pollutants. The persistence of diverse coral assemblages at Tung Ping Chau may contribute to increased pathogen resistance while the eutrophication of Tolo Harbour and disappearance of coral communities [27] may have reduced this important ecosystem service. More direct links between human activity and pollution stress can be inferred from our antibiotic resistance analysis which shows an increase in the dominant resistance mechanisms and abundance of ARGs. It is well known that efflux pumps are the predominant resistance mechanism in sediments [17], and our results further provide the direct evidence that an increase in efflux pump-related genes is correlated to an increase in pollution [46]. These data suggest the increased capability of microbes to reduce intracellular concentrations of antibiotics, heavy metals, or other toxins/environmental stresses [17] in polluted sediments. In other words, the increasing efflux pump mechanisms in the microbes would greatly contribute to antibiotic resistance and further presented a growing threat to antibiotic therapy and a major challenge for antibiotic development.

## Conclusions

Our investigation of the microbiome composition and functionality in marine sediments revealed that microbe communities are likely influenced by human activity and pollution discharge. Through our analysis, we also identified several microbes within the *Planctomycetes* phylum that may serve as useful bio-indicators of water quality. The relationships among sediment microbial communities and water quality presented here set the stage for a valuable reference database for environmental assessments and public policy. Therefore, we strive for the continuous sampling of these as well as additional sites in Hong Kong to provide new insight into the interactions between humans and coastal marine ecosystems.

## Materials and methods

### Sampling sites and sample collection

Sampling was conducted in August 2016 from four field sites (Centre Island (CI), Che Lei Pai (CLP), Port Island (PI), and Tung Ping Chau (TPC)) in Tolo Harbour. At each sampling site, 50 g of sediment from the top 10 cm were collected via scuba using sterile 15-mL falcon tubes and were further divided into three aliquots as replicates (Fig. 1). Within minutes after sampling, sediment samples were flash frozen in liquid nitrogen and stored at − 80 °C for DNA extraction and metagenomic shotgun sequencing.

### Pollution data collection

Seven geochemical indicators (zinc (Zn), lead (Pb), copper (Cu), chemical oxygen demand (COD), arsenic (As), total volatile solid (TVS), and ammonia nitrogen (NH_3_-N)) were obtained from the Hong Kong Environmental Protection Department’s extensive periodic water and sediment monitoring database [47] (Additional file 1: Table S1) representing the environmental pollution in the sediment including heavy metals and organic matter. These data were obtained from the sediment samples collected in August 2016 at the benthic sediment sampling locations. Pollution data were normalized using the minimum-maximum normalization procedure for further analyses (Additional file 6: Figure S1).

### DNA extraction and metagenomic sequencing

DNA from the sediment samples was extracted using the MoBio PowerSoil DNA Isolation Kit (MoBio, Carlsbad, CA) following the manufacturer’s instructions. DNA concentrations (2.41 ± 1.25 μg) were verified using the Qubit Fluorometer and DNA quality was checked via agarose gel electrophoresis (using Takara λ-Hind III digest and the Tiangen D2000 marker) prior to library construction. DNA libraries were constructed using the Nextera XT kit. Metagenomic shotgun sequencing was then performed on an Illumina HiSeq 1500 (101 bp PE) platform. The raw sequence data were uploaded into the Sequence Read Archive of NCBI (accession numbers SRR8361706-17).

### Taxonomic profiling

The raw sequencing paired-end reads passed standard quality control after the adapter regions and low-quality reads were removed, as per Li et al. [48]. The filtered reads were mapped to the nonredundant database using DIAMOND [49] and the default settings. The aligned reads were filtered using an *e*-value < 1e−10 and a 95% cutoff. The lowest common ancestor (LCA) algorithm was implemented with the LCA mapper from mtools of MEGAN5 [50] for the taxonomic assignment of each aligned read. The relative abundances of each taxa were further distilled from the LCA results for each taxonomic level. Prokaryotic community profiles were constructed at phylum, family, and genus levels for further statistical analysis. Additionally, potential pathogenic species and genera were taxonomically identified using three publicly available lists of putative pathogens [23–25] and further summarized to genus level for statistical comparisons. The organisms appearing on the list were regarded as opportunistic pathogens and the species containing at least one opportunistic pathogenic strain were marked as putative pathogenic species.

### Microbial community composition

Alpha diversity indices detailing microbial community composition within each sample was calculated using vegan [51] in R. Both the Shannon and Simpson indices were used for alpha diversity evaluation based on the relative abundance of each taxonomic level. For estimating community dissimilarities, both Bray-Curtis and Euclidean distances were calculated by phyloseq [52] and vegan [51] based on the relative abundance of each taxon at different levels.

### Estimation of species cellular replication rate

To estimate the replication rate of the 30 most abundant species, an in-house genome database was constructed manually by obtaining the draft or complete genomes of the target species (either contig or scaffold) from the NCBI Genome database. The high-quality metagenomic reads were mapped to the in-house genome database by Bowtie2 [53]. The mapped results of each target species were further manipulated with SAMtools [54] and were used for measuring replication rates with iRep [21].

### Functional annotation

From each sample’s filtered sequencing data, one million reads were subsampled randomly and were aligned to the KEGG Orthology (KO) database [55] built by KOBAS 2.0 [56] using DIAMOND BLASTX (−e 1e−10, best hits reserved). The identified KO genes were further annotated into different pathways based on predefined collections in the KEGG database and were quantified by read counts. The prokaryotic taxonomic profiles of the 25 most abundant families among samples and the pathway profile were integrated to quantify taxa-specific contributions to functional shifts using FishTaco [22].

### Antibiotic resistance genes (ARGs)

Subsampled reads were mapped against an in-house ARG database based on CARD [57], ARDB [58], and ResFams [59] using BLASTX. Aligned reads were filtered (best hit from BLASTX, identity > 70% and coverage > 70%), classified into different ARG mechanisms, and further annotated into ARG families using ResFams. The abundance of ARG genes was calculated as RPKM (reads per million mapped reads).

### Data analysis

Three replicate samples were analyzed. Statistical comparisons of prokaryotic taxonomic profiles, potential pathogens, and ARGs (including clinically important ARGs) were performed by using Kruskal-Wallis tests between the four sampling sites and Student’s *t* tests between each pairwise site in R. The Benjamini-Hochberg FDR correction [60] was applied to adjust the *P* value for multiple *t* test comparisons in R (Additional file 4). We performed principal component analysis (PCA) on the relative abundance of taxonomic profiles to evaluate the community dissimilarities using the vegan and ape [61] packages in R (Additional file 11). The pollution parameters were served as fitted vectors in the above multidimensional scaling for testing the correlation between community distributions and pollution levels. Adonis from vegan package in R was used as PERMANOVA test to evaluate the significance of a variable in determining variation of distances (the number of permutations were set as 999). The homogeneity of dispersions test was calculated using PERMDISP from vegan package in R. Pearson’s correlation coefficients were calculated between the taxa-based and KO function-based profiles using FishTaco. Spearman correlations were further performed between taxonomic abundance and KO pathways among the four sampling sites based on the FishTaco results using the correlation function in R.

### Data visualization

Packages including ggplot2 and gplots in R and matplotlib in Python were used for visualization purposes.

## Additional files

Additional file 1: **Table S1.** Representative water quality data from the Hong Kong Environmental Protection Department. (CSV 264 bytes)

Additional file 2: **Table S2.** Significant statistical comparison result on the top 50 abundant prokaryotes at phylum/family/genus levels. (XLSX 46 kb)

Additional file 3: **Table S3.** Significantly enriched KEGG pathways in Centre Island comparing to Tung Ping Chau. (XLSX 9 kb)

Additional file 4: **Table S4.** Taxonomic significant statistical comparison result between Centre Island and Tung Ping Chau with FDR correction. (XLSX 51 kb)

Additional file 5: **Table S5.** PERMANOVA and PERMDISP results on microbial communities among the four sampling sites. (XLSX 35 kb)

Additional file 6: **Figure S1.** Representative water quality data among the four sampling sites. (PDF 4 kb)

Additional file 7: **Figure S2.** The comparison of alpha diversity in the Shannon index among sampling sites. (PDF 4 kb)

Additional file 8: **Figure S3.** Comparisons of bacterial replication rate among the four sampling sites. (PDF 8 kb)

Additional file 9: **Figure S4.** Pearson’s correlation coefficients between the taxa-based and KO function-based profiles for the significantly different pathways. (PDF 5 kb)

Additional file 10: **Figure S5.** Spearman’s correlation results between the correlated pathways and families among the four sampling sites. (PDF 31 kb)

Additional file 11: **File S1.** Statistical analysis scripts performed in R. (PDF 72 kb)

## Data Availability

The raw Illumina sequence data of metagenomic data have been deposited in the sequence read archive (SRA accession: SRR8361706-17) at NCBI under Bioproject accession #PRJNA511137. The scripts for all statistical analysis are available as Additional file 11.

## Abbreviations

ARGs: Antibiotic resistance genes
COD: Chemical oxygen demand
iRep: Index of replication
KEGG: Kyoto Encyclopedia of Genes and Genomes
LCA: Lowest common ancestor
PCA: Principle component analyses
PRD: Pearl river delta
RPKM: Reads per million mapped reads
RPM: Reads per million mapped reads
SRM: Sulfur-reducing microbe
TVS: Total volatile solid
