# Supplementary figures and images for: A pollution gradient contributes to the taxonomic, functional, and resistome diversity of microbial communities in marine sediments
Source: Microbiome. 2019 Jul 15;7:104. doi: 10.1186/s40168-019-0714-6 (PMC6632204; doi:10.1186/s40168-019-0714-6)

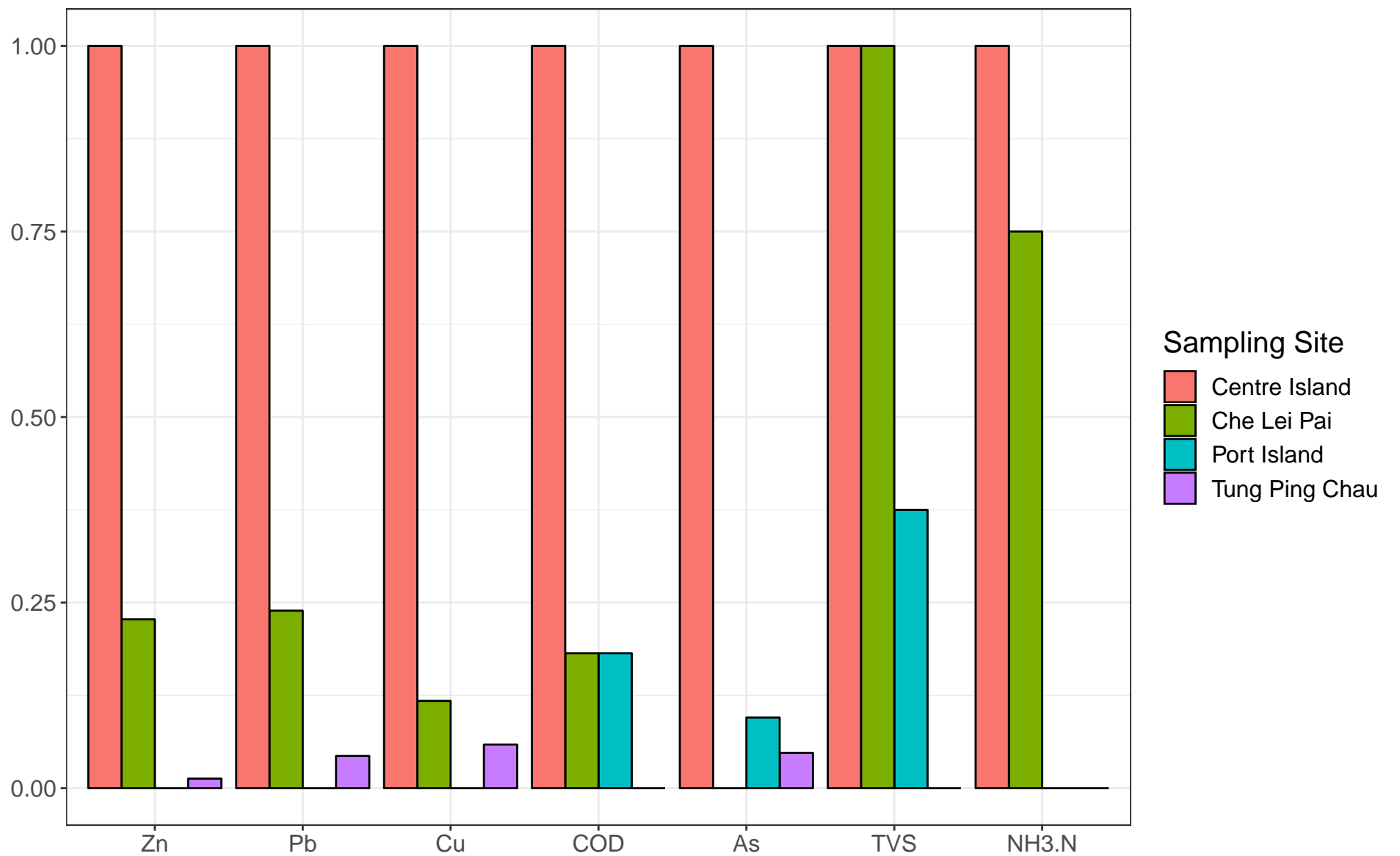

Supplement: Supplementary file 6 — Figure S1. Representative water quality data among the four sampling sites. (PDF 4 kb) [file 40168_2019_714_MOESM6_ESM.pdf]

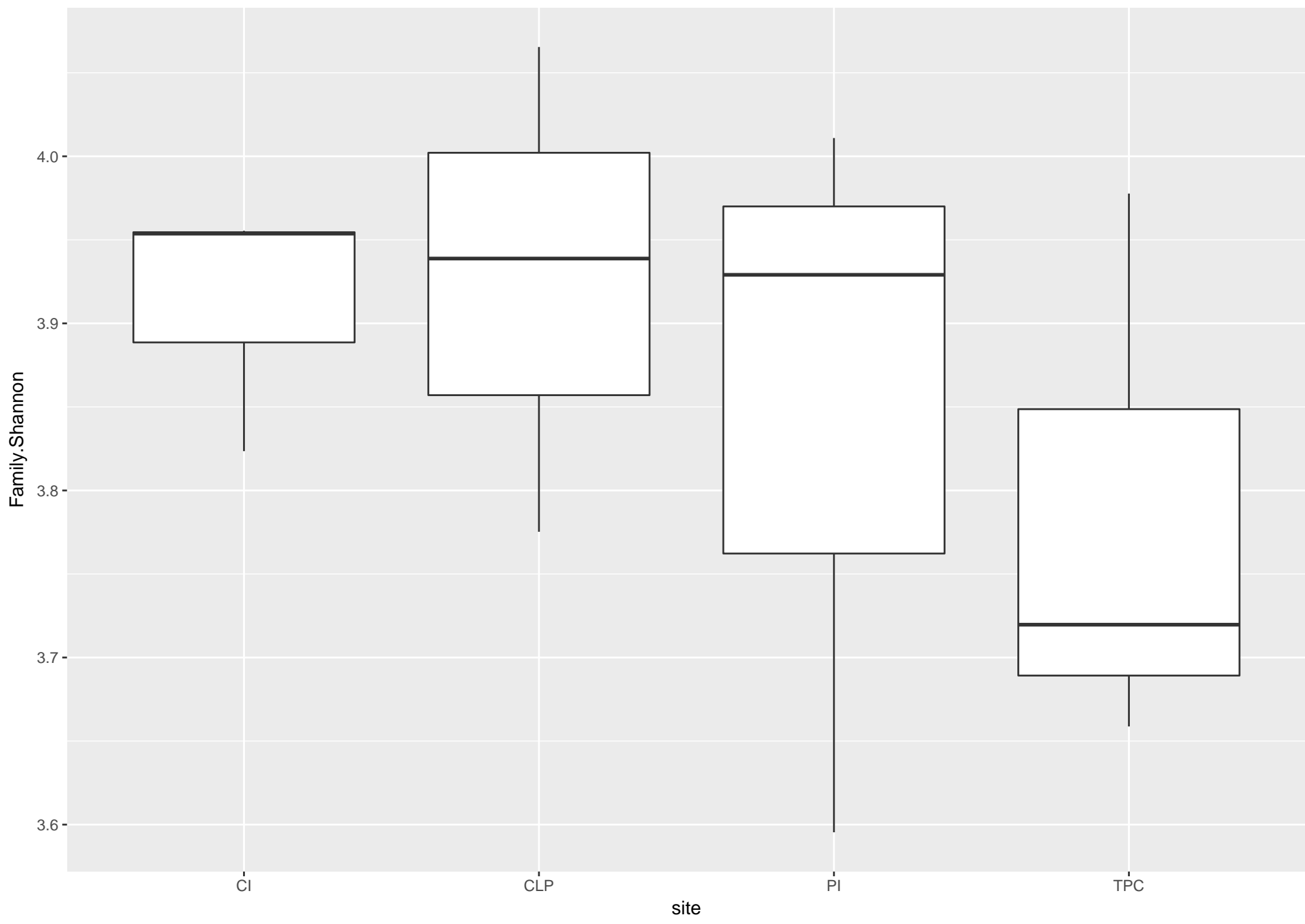

Supplement: Supplementary file 7 — Figure S2. The comparison of alpha diversity in the Shannon index among sampling sites. (PDF 4 kb) [file 40168_2019_714_MOESM7_ESM.pdf]

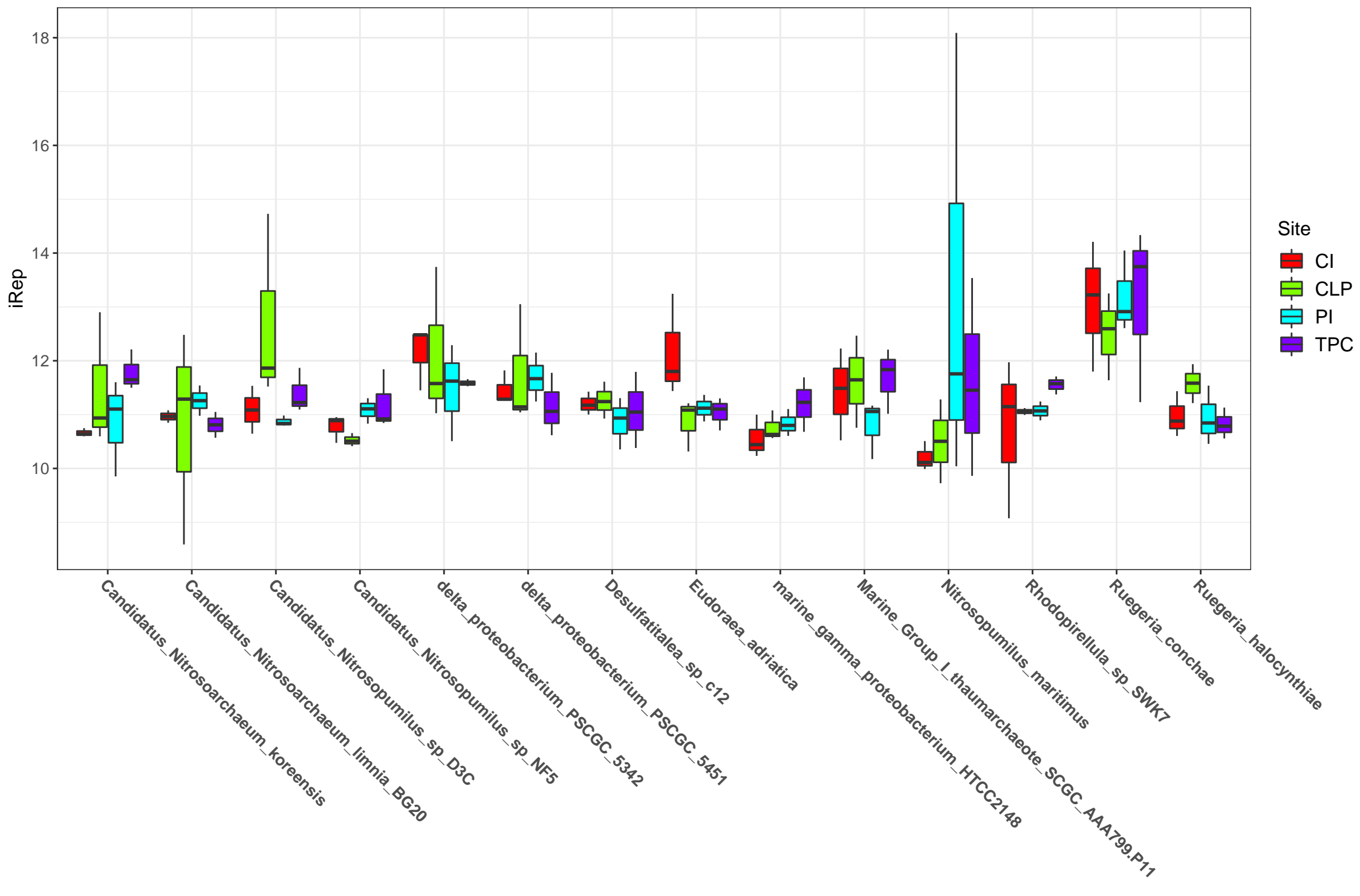

Supplement: Supplementary file 8 — Figure S3. Comparisons of bacterial replication rate among the four sampling sites. (PDF 8 kb) [file 40168_2019_714_MOESM8_ESM.pdf]

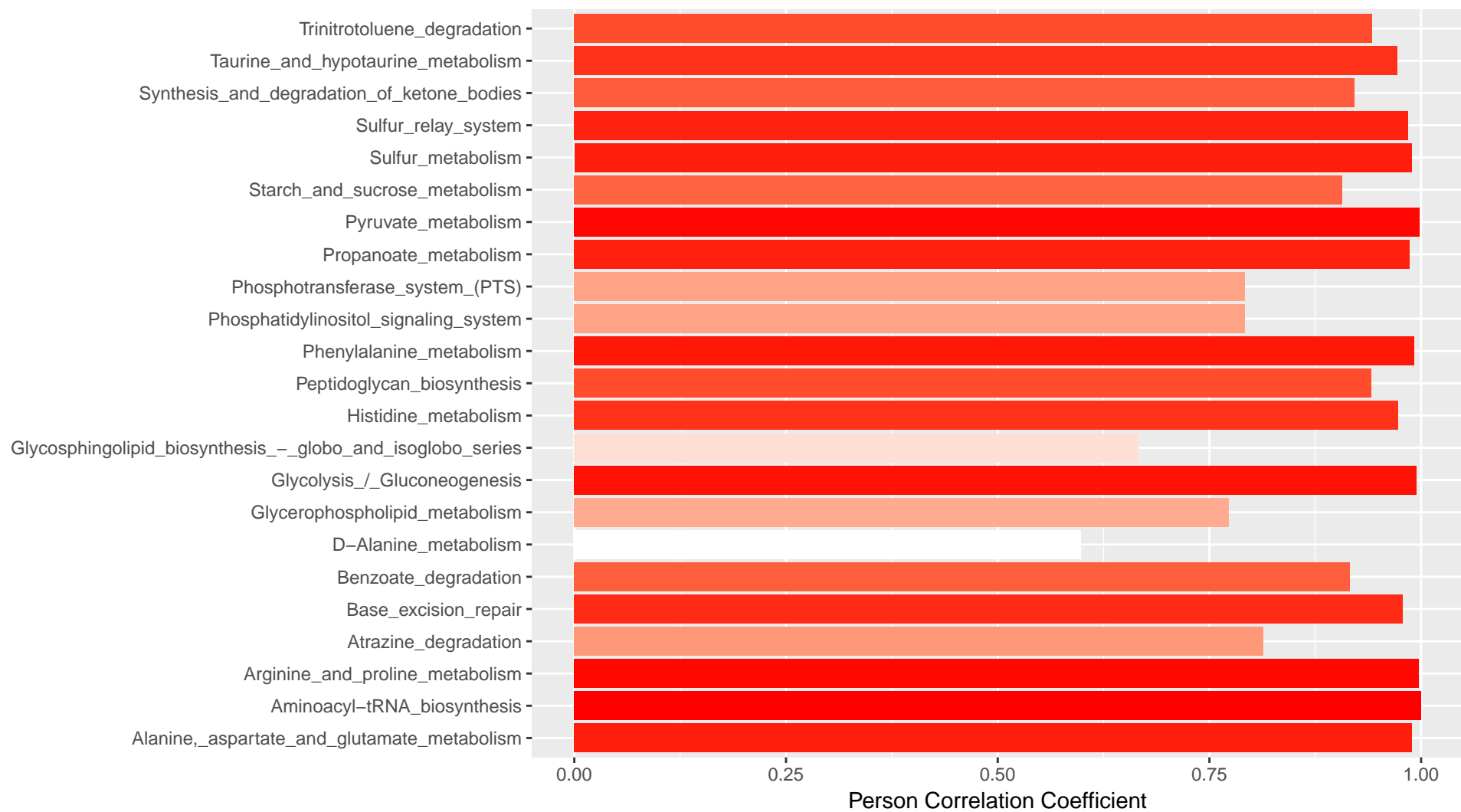

Supplement: Supplementary file 9 — Figure S4. Pearson’s correlation coefficients between the taxa-based and KO function-based profiles for the significantly different pathways. (PDF 5 kb) [file 40168_2019_714_MOESM9_ESM.pdf]

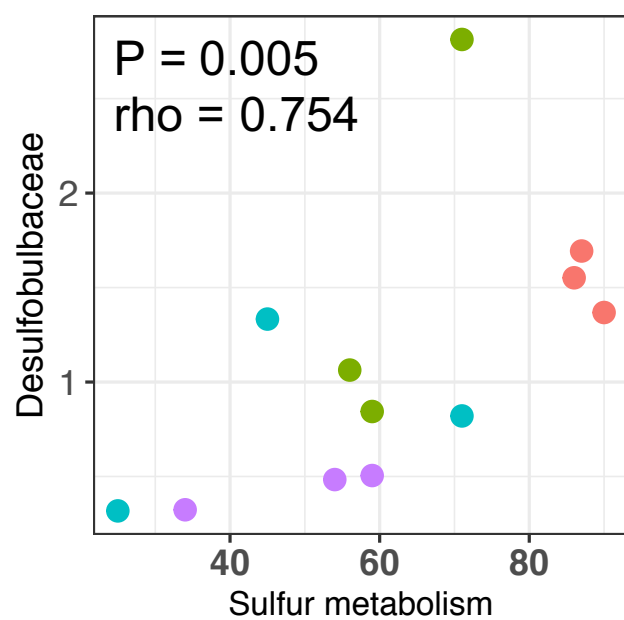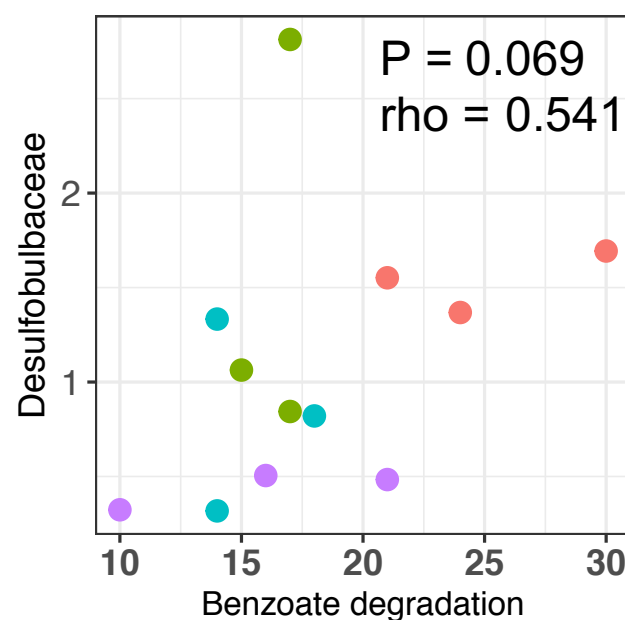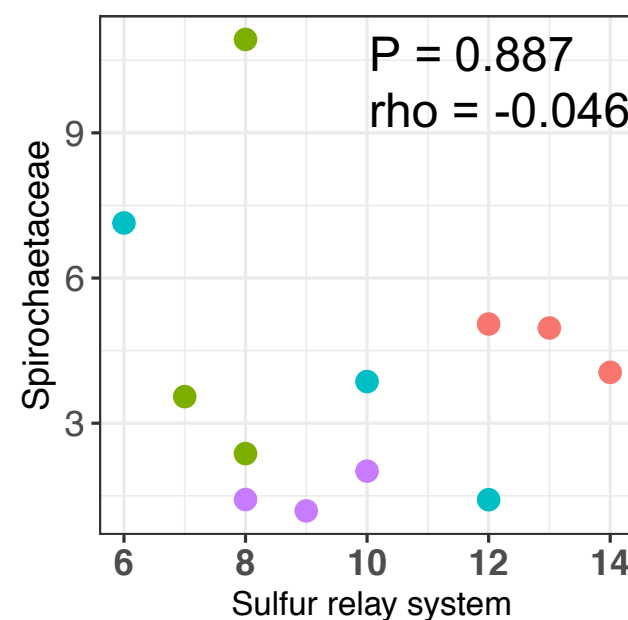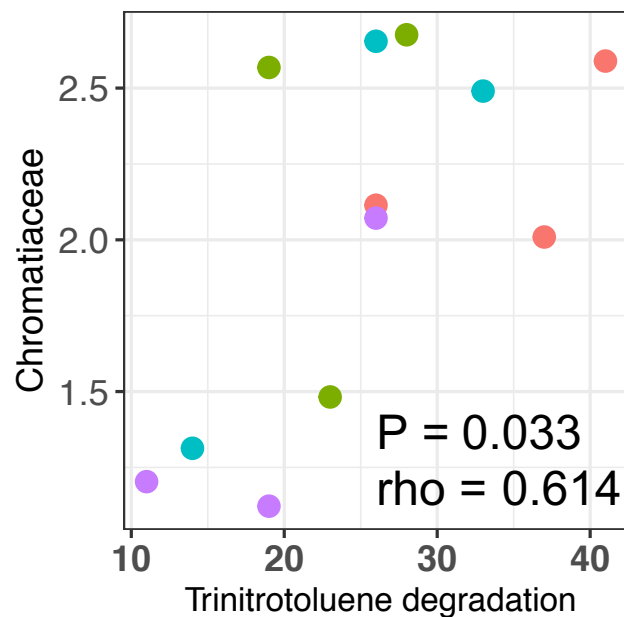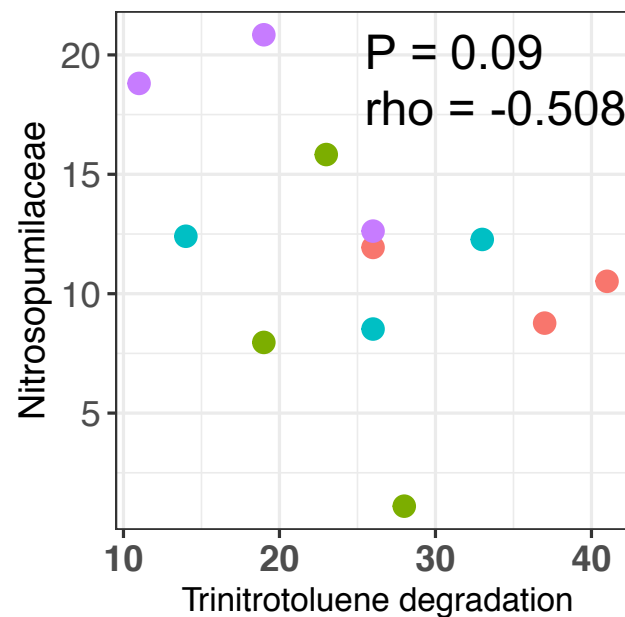

Supplement: Supplementary file 10 — Figure S5. Spearman’s correlation results between the correlated pathways and families among the four sampling sites. (PDF 31 kb) [file 40168_2019_714_MOESM10_ESM.pdf]
