# Supplementary material for: A pollution gradient contributes to the taxonomic, functional, and resistome diversity of microbial communities in marine sediments
Source: Microbiome. 2019 Jul 15;7:104. doi: 10.1186/s40168-019-0714-6 (PMC6632204; doi:10.1186/s40168-019-0714-6)
Supplement: Supplementary file 11 — File S1. Statistical analysis scripts performed in R. (PDF 72 kb) [file 40168_2019_714_MOESM11_ESM.pdf]

April 17, 2019

The results below are generated from an R script.

```
## Loading required package
library(vegan)
library(ggplot2)
library(reshape)
library(dplyr)

## Inputting the OTU table and pollution concentration data
phylum<-"phylum.otu"
pollution<-"pollution.csv"
genus<-"genus.csv"

######Statistical Comparisons
###Kruskal-Wallis test
taxon<-read.csv(phylum,row.names = 1,sep = "\t")
group<-c("CI","CI","CI","CLP","CLP","CLP","PI","PI","PI","TPC","TPC","TPC")
taxon<-rbind(group,taxon)
rownames(taxon)[1]<-"group"
taxon<-t(taxon)
taxon<-as.data.frame(taxon)

kruskal_test<-function(sample){
  x<-as.factor(taxon$group)
  y<-kruskal.test(sample,x)
  z<-y$p.value
}
p<-apply(taxon[2:length(taxon)], 2, kruskal_test)
significant_p<-p[which(p<=0.05)]
adjust_p=p.adjust(p,method="fdr",n=length(p)) ###FDR correction
kruskal_result<-cbind(p,adjust_p)
#write.csv(kruskal_result,"kruskal_result.csv")
head(kruskal_result)

##                          p  adjust_p
## Proteobacteria 0.24794893 0.5578851
## Thaumarchaeota 0.93273106 0.9327311
## Bacteroidetes  0.54622778 0.6742938
## Planctomycetes 0.02998054 0.5573567
## Cyanobacteria  0.83839872 0.9054706
## Spirochaetes   0.12067680 0.5573567

###Student's t-tests
CI_TPC<-taxon[which((taxon$group=="CI")|(taxon$group=="TPC")),] ###Take comparison between Cen-
tre Island and Tung Ping Chau for example
t_test<-function(sample){
```

```
  x<-as.numeric(sample[1:3])
  y<-as.numeric(sample[4:6])
  z<-t.test(x,y)
  w<-z$p.value
}
p<-apply(CI_TPC[2:length(CI_TPC)], 2, t_test)
significant_p<-p[which(p<=0.05)]
significant_p ###significant result
```

```
##                Planctomycetes               Spirochaetes
##                   0.013656950                0.001246202
##                    Firmicutes               Nitrospinae
##                   0.043713455                0.038414224
##              Ignavibacteriae Thermodesulfobacteria <phylum>
##                   0.004415479                0.035381708
##              Armatimonadetes                 Omnitrophica
##                   0.037223075                0.001065630
##                  Acetothermia
##                   0.018350681
```

```
adjust_p=p.adjust(p,method="fdr",n=length(p)) ###FDR correction
t_test_result<-cbind(p,adjust_p)
#write.csv(t_test_result,"t_test_result.csv")
head(t_test_result)
```

```
##                          p    adjust_p
## Proteobacteria 0.349013212 0.50547626
## Thaumarchaeota 0.083236215 0.34575043
## Bacteroidetes  0.611956428 0.75103743
## Planctomycetes 0.013656950 0.18436882
## Cyanobacteria  0.445243475 0.57245590
## Spirochaetes   0.001246202 0.03364745
```

```
######Example for visualization
###Setting color parameters
manualcolors<-c('forestgreen', 'red2', 'orange', 'cornflowerblue',
                'magenta', 'darkolivegreen4',
                'indianred1', 'tan4', 'darkblue',
                'mediumorchid1','firebrick4',  'yellowgreen', 'lightsalmon', 'tan3',
                "tan1",'darkgray', 'wheat4', '#DDAD4B', 'chartreuse', 'seagreen1',
                'moccasin', 'mediumvioletred', 'seagreen','cadetblue1',
                "darkolivegreen1" ,"tan2" ,   "tomato3" , "#7CE3D8","gainsboro","black")
color2<-as.factor(c("CI","CLP","PI","TPC"))
col.rainbow <- rainbow(nlevels(color2))
col.topo <- topo.colors(nlevels(color2))
col.terrain <- terrain.colors(nlevels(color2))
palette(col.rainbow)

taxon<-read.csv(phylum,row.names = 1,sep = "\t")
taxon<-taxon[1:10,] ###Take the top ten abundant phylum for visualization
taxon<-t(taxon)
taxon<-as.data.frame(taxon)
taxon$Site<-c("CI","CI","CI","CLP","CLP","CLP","PI","PI","PI","TPC","TPC","TPC")
taxon$SampleID<-rownames(taxon)
taxon<-melt(taxon)
```

```
## Using Site, SampleID as id variables

barchart1<-ggplot(taxon,aes(x=SampleID,y=value,fill=variable))+
  geom_bar(stat="identity",position = "fill")+
  theme_bw()+
  scale_fill_manual(name="Phylum",values = manualcolors)+
  labs(x="",y="Percentage")+
  theme(axis.text.x = element_text(angle=90,hjust = 1,vjust = 0.5,size = 12,face = "bold"),
        legend.text=element_text(size=12),
        legend.title = element_text(size=12),
        axis.text.y = element_text(size=10),
        axis.title.y = element_text(size=12)
  )
barchart1
```

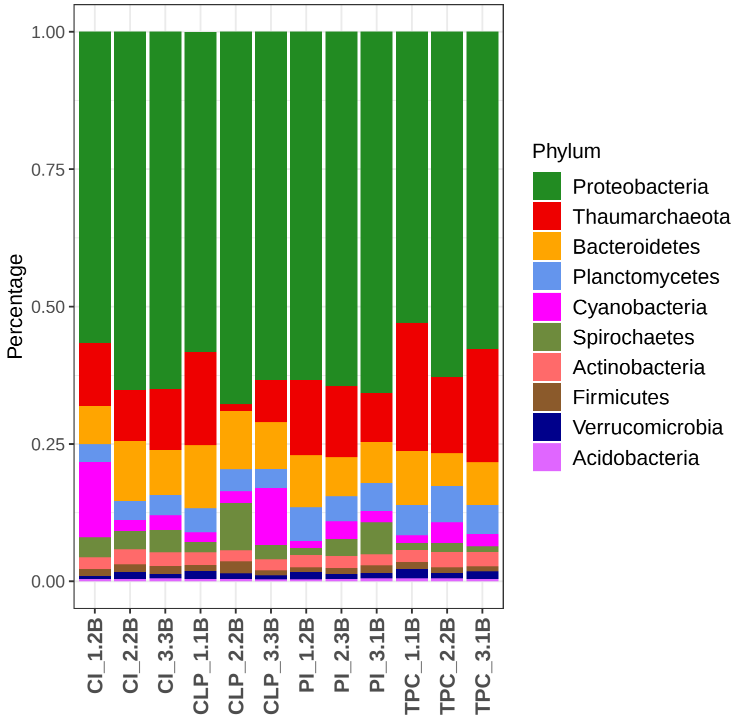


```
######Diversity Analyses
###alpha-diversity
taxon<-read.csv(phylum,row.names = 1,sep = "\t")
taxon<-t(taxon)

shannon_diversity<-as.data.frame(diversity(taxon))
colnames(shannon_diversity)="Shannon_diveristy"
simpson_diversity<-as.data.frame(diversity(taxon,index="simpson"))
colnames(simpson_diversity)="Simpson_diveristy"
alpha_diversity<-merge(shannon_diversity,simpson_diversity,by="row.names",all.x=TRUE)
row.names(alpha_diversity)<-alpha_diversity$Row.names
alpha_diversity$Row.names<-NULL
alpha_diversity

##          Shannon_diveristy Simpson_diveristy
```

```
## CI_1.2B           1.586628          0.6557702
## CI_2.2B           1.456493          0.5763483
## CI_3.3B           1.462565          0.5779670
## CLP_1.1B          1.544652          0.6381458
## CLP_2.2B          1.430298          0.5540924
## CLP_3.3B          1.454116          0.5904911
## PI_1.2B           1.397794          0.5840128
## PI_2.3B           1.429276          0.5762481
## PI_3.1B           1.445128          0.5686715
## TPC_1.1B          1.589569          0.6719207
## TPC_2.2B          1.461714          0.5935687
## TPC_3.1B          1.492628          0.6314416

#write.csv(alpha_diversity,"alpha_diversity.csv")

###beta-diversity
taxon<-as.matrix(taxon)
bray_curtis<-vegdist(taxon,method="bray")
bray_curtis<-as.matrix(bray_curtis)
bray_curtis

##             CI_1.2B    CI_2.2B    CI_3.3B   CLP_1.1B   CLP_2.2B   CLP_3.3B    PI_1.2B
## CI_1.2B  0.00000000 0.14243336 0.11457641 0.13849894 0.21919890 0.08966424 0.15836547
## CI_2.2B  0.14243336 0.00000000 0.03794998 0.09529220 0.09714764 0.08384420 0.07320648
## CI_3.3B  0.11457641 0.03794998 0.00000000 0.10760115 0.11036484 0.08232267 0.07263028
## CLP_1.1B 0.13849894 0.09529220 0.10760115 0.00000000 0.18125072 0.14814710 0.07319393
## CLP_2.2B 0.21919890 0.09714764 0.11036484 0.18125072 0.00000000 0.15024662 0.15812929
## CLP_3.3B 0.08966424 0.08384420 0.08232267 0.14814710 0.15024662 0.00000000 0.10741143
## PI_1.2B  0.15836547 0.07320648 0.07263028 0.07319393 0.15812929 0.10741143 0.00000000
## PI_2.3B  0.11499053 0.06219236 0.03652288 0.09659672 0.13965852 0.08602924 0.05521027
## PI_3.1B  0.14201303 0.05347726 0.04074648 0.13047383 0.09071077 0.09211864 0.08953825
## TPC_1.1B 0.18563013 0.16500423 0.16490173 0.08079341 0.24883958 0.20896978 0.11412378
## TPC_2.2B 0.13530851 0.09817490 0.07520803 0.10193168 0.18197939 0.10642078 0.04589975
## TPC_3.1B 0.14565470 0.13795769 0.11924069 0.05993236 0.22264393 0.16254336 0.08758420
##             PI_2.3B    PI_3.1B   TPC_1.1B   TPC_2.2B   TPC_3.1B
## CI_1.2B  0.11499053 0.14201303 0.18563013 0.13530851 0.14565470
## CI_2.2B  0.06219236 0.05347726 0.16500423 0.09817490 0.13795769
## CI_3.3B  0.03652288 0.04074648 0.16490173 0.07520803 0.11924069
## CLP_1.1B 0.09659672 0.13047383 0.08079341 0.10193168 0.05993236
## CLP_2.2B 0.13965852 0.09071077 0.24883958 0.18197939 0.22264393
## CLP_3.3B 0.08602924 0.09211864 0.20896978 0.10642078 0.16254336
## PI_1.2B  0.05521027 0.08953825 0.11412378 0.04589975 0.08758420
## PI_2.3B  0.00000000 0.05425704 0.15370657 0.04318070 0.09927212
## PI_3.1B  0.05425704 0.00000000 0.18414363 0.09212342 0.13458514
## TPC_1.1B 0.15370657 0.18414363 0.00000000 0.14567323 0.06423763
## TPC_2.2B 0.04318070 0.09212342 0.14567323 0.00000000 0.08830326
## TPC_3.1B 0.09927212 0.13458514 0.06423763 0.08830326 0.00000000

#write.csv(bray_curtis,"bray_curtis.csv")

######Principal component analyses
taxon<-read.csv(genus,row.names = 1)
taxon<-t(taxon)
taxan<-as.data.frame(taxon)
pollution<-read.csv(pollution,row.names = 1)
```

```
row.names(pollution)<-row.names(taxon)

###Setting color parameters
color2 <- as.factor(c("CI","CI","CI","CLP","CLP","CLP","PI","PI","PI","TPC","TPC","TPC"))
col.rainbow <- rainbow(nlevels(color2))
col.topo <- topo.colors(nlevels(color2))
col.terrain <- terrain.colors(nlevels(color2))
palette(col.rainbow)

###Visualizing PCA
pc <- prcomp(taxon)
fig = sprintf("%s.pca.pdf","genus")
title = sprintf("Principle Component Analyses (%s)", "genus")
#pdf(fig)
plot(pc$x[,1:2], xlab = "PC1", ylab = "PC2", type="p", cex=1.5, pch=16, col=color2,main = title)
legend("bottomleft", pch=16, cex=1, pt.cex=1.5,col=1:nlevels(color2), legend=levels(color2))
text(pc$x[,1:2], labels=row.names(it), cex=1, pos=3)
ef2<-envfit(pc, pollution, perm=999)
plot(ef2,cex=0.7,col="blue",p.max = 0.05)
ef<-envfit(pc,taxon,perm=999)
plot(ef,cex=0.7,col="grey",p.max = 0.01)
abline(h=0,lty=2)
abline(v=0,lty=2)
```

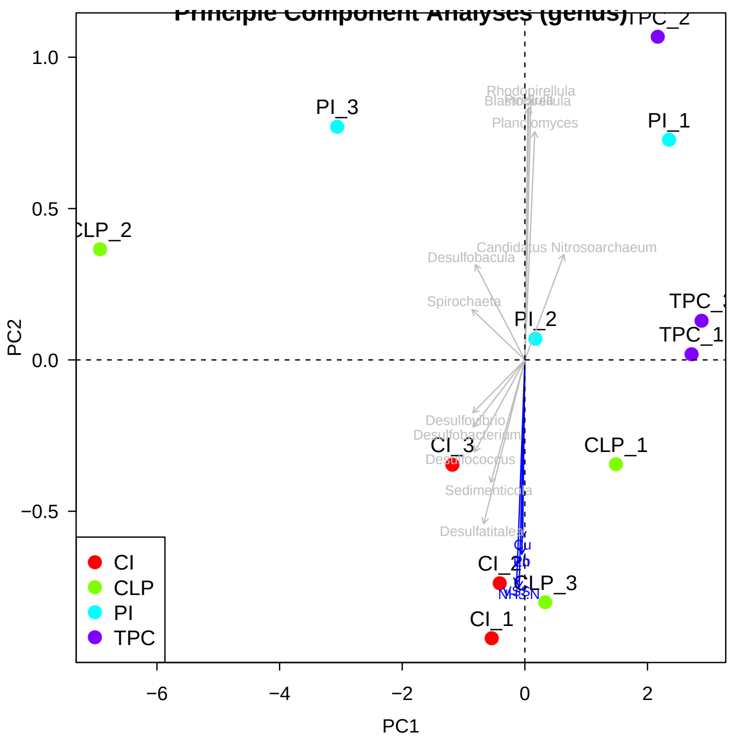


```
###PERMANOVA & PERMDISP
adonis(taxon ~ Zn, data= pollution, permutations = 999)

##
## Call:
```

```
## adonis(formula = taxon ~ Zn, data = pollution, permutations = 999)
##
## Permutation: free
## Number of permutations: 999
##
## Terms added sequentially (first to last)
##
##           Df SumsOfSqs  MeanSqs F.Model      R2 Pr(>F)
## Zn         1   0.12229 0.122285  2.9875 0.23003  0.099 .
## Residuals 10   0.40932 0.040932         0.76997
## Total     11   0.53160                  1.00000
## ---
## Signif. codes:  0 '***' 0.001 '**' 0.01 '*' 0.05 '.' 0.1 ' ' 1
```

```
adonis(taxon ~ Pb, data= pollution, permutations = 999)
```

```
##
## Call:
## adonis(formula = taxon ~ Pb, data = pollution, permutations = 999)
##
## Permutation: free
## Number of permutations: 999
##
## Terms added sequentially (first to last)
##
##           Df SumsOfSqs  MeanSqs F.Model      R2 Pr(>F)
## Pb         1   0.11782 0.117824  2.8475 0.22164  0.093 .
## Residuals 10   0.41378 0.041378         0.77836
## Total     11   0.53160                  1.00000
## ---
## Signif. codes:  0 '***' 0.001 '**' 0.01 '*' 0.05 '.' 0.1 ' ' 1
```

```
adonis(taxon ~ Cu, data= pollution, permutations = 999)
```

```
##
## Call:
## adonis(formula = taxon ~ Cu, data = pollution, permutations = 999)
##
## Permutation: free
## Number of permutations: 999
##
## Terms added sequentially (first to last)
##
##           Df SumsOfSqs  MeanSqs F.Model      R2 Pr(>F)
## Cu         1   0.09332 0.093317  2.1291 0.17554  0.133
## Residuals 10   0.43829 0.043829         0.82446
## Total     11   0.53160                  1.00000
```

```
adonis(taxon ~ COD, data= pollution, permutations = 999)
```

```
##
## Call:
## adonis(formula = taxon ~ COD, data = pollution, permutations = 999)
##
## Permutation: free
```

```
## Number of permutations: 999
##
## Terms added sequentially (first to last)
##
##           Df SumsOfSqs  MeanSqs F.Model      R2 Pr(>F)
## COD        1   0.12479 0.124786  3.0674 0.23474  0.076 .
## Residuals 10   0.40682 0.040682         0.76526
## Total     11   0.53160                  1.00000
## ---
## Signif. codes:  0 '***' 0.001 '**' 0.01 '*' 0.05 '.' 0.1 ' ' 1

adonis(taxon ~ As, data= pollution, permutations = 999)

##
## Call:
## adonis(formula = taxon ~ As, data = pollution, permutations = 999)
##
## Permutation: free
## Number of permutations: 999
##
## Terms added sequentially (first to last)
##
##           Df SumsOfSqs  MeanSqs F.Model     R2 Pr(>F)
## As         1   0.07793 0.077933  1.7178 0.1466  0.166
## Residuals 10   0.45367 0.045367         0.8534
## Total     11   0.53160                  1.0000

adonis(taxon ~ VSS, data= pollution, permutations = 999)

##
## Call:
## adonis(formula = taxon ~ VSS, data = pollution, permutations = 999)
##
## Permutation: free
## Number of permutations: 999
##
## Terms added sequentially (first to last)
##
##           Df SumsOfSqs  MeanSqs F.Model      R2 Pr(>F)
## VSS        1   0.23753 0.237526   8.077 0.44681  0.005 **
## Residuals 10   0.29408 0.029408         0.55319
## Total     11   0.53160                  1.00000
## ---
## Signif. codes:  0 '***' 0.001 '**' 0.01 '*' 0.05 '.' 0.1 ' ' 1

adonis(taxon ~ NH3.N, data= pollution, permutations = 999)

##
## Call:
## adonis(formula = taxon ~ NH3.N, data = pollution, permutations = 999)
##
## Permutation: free
## Number of permutations: 999
##
## Terms added sequentially (first to last)
```

```
##
##           Df SumsOfSqs  MeanSqs F.Model      R2 Pr(>F)
## NH3.N      1   0.18711 0.187108  5.4314 0.35197  0.024 *
## Residuals 10   0.34449 0.034449         0.64803
## Total     11   0.53160                  1.00000
## ---
## Signif. codes:  0 '***' 0.001 '**' 0.01 '*' 0.05 '.' 0.1 ' ' 1

bray_curtis<-vegdist(taxon)
group<-c("CI","CI","CI","CLP","CLP","CLP","PI","PI","PI","TPC","TPC","TPC")
mod<-betadisper(bray_curtis,group)
mod

##
##  Homogeneity of multivariate dispersions
##
## Call: betadisper(d = bray_curtis, group = group)
##
## No. of Positive Eigenvalues: 8
## No. of Negative Eigenvalues: 3
##
## Average distance to median:
##      CI     CLP      PI     TPC
## 0.05379 0.19746 0.14773 0.06416
##
## Eigenvalues for PCoA axes:
##  PCoA1  PCoA2  PCoA3  PCoA4  PCoA5  PCoA6  PCoA7  PCoA8
## 0.4072 0.0897 0.0216 0.0109 0.0080 0.0038 0.0029 0.0003

######Spearman Correlation Analyses
###Between significantly correlated function and speices among four sampling sites based on the Fish-
Taco results
fun1<-c(86,	90,	87,	59,	71,	56,	25,	71,	45,	54,	59,	34)# Sulfur_metabolism
fun2<-c(21,	24,	30,	17,	17,	15,	14,	18,	14,	21,	16,	10) # Benzoate degradation
fun3<-c(13,	14,	12,	8,	8,	7,	12,	10,	6,	8,	10,	9) # Sulfur relay system
fun4<-c(26,	37,	41,	23,	28,	19,	14,	33,	26,	19,	26,	11) # Trinitrotoluene degradation

tax1<-c(1.551764944,	1.367980445,	1.693402047,	0.843973072,	2.813094691,	1.063352128,
	0.317951574,	0.820540512,	1.333140774,	0.483673149,	0.505020095,	0.323748049) # Desul-
fobulbaceae
tax2<-c(4.967774466,	4.051488098,	5.049720549,	2.371944813,	10.92919483,	3.549256128,
	1.419854701,	3.860590284,	7.13780602,	1.422893555,	2.010296088,	1.188581984) # Spirochaetaceae
tax3<-c(2.113953879,	2.009308701,	2.589097772,	1.482141236,	2.675700505,	2.567932788,
	1.313048352,	2.49008807,	2.654329595,	1.122269252,	2.071636084,	1.203138097) # Chromatiaceae
tax4<-c(11.93733943,	8.767733566,	10.51898091,	15.82665692,	1.102024411,	7.959814529,
	12.40539883,	12.27230441,	8.520248601,	20.84267021,	12.62061024,	18.80599712) # Ni-
trosopumilaceae

cor.test(fun1, tax1,method="spearman")##p = 0.00458 rho = 0.7543906

## Warning in cor.test.default(fun1, tax1, method = "spearman"): Cannot compute exact p-value with ties

##
##  Spearman's rank correlation rho
##
```

```
## data:  fun1 and tax1
## S = 70.244, p-value = 0.00458
## alternative hypothesis: true rho is not equal to 0
## sample estimates:
##       rho
## 0.7543906

cor.test(fun2, tax1,method="spearman")##p = 0.06913 rho = 0.5413081

## Warning in cor.test.default(fun2, tax1, method = "spearman"): Cannot compute exact p-value with ties

##
##  Spearman's rank correlation rho
##
## data:  fun2 and tax1
## S = 131.19, p-value = 0.06913
## alternative hypothesis: true rho is not equal to 0
## sample estimates:
##       rho
## 0.5413081

cor.test(fun3, tax2,method="spearman")##p = 0.8873 rho = -0.04593898

## Warning in cor.test.default(fun3, tax2, method = "spearman"): Cannot compute exact p-value with ties

##
##  Spearman's rank correlation rho
##
## data:  fun3 and tax2
## S = 299.14, p-value = 0.8873
## alternative hypothesis: true rho is not equal to 0
## sample estimates:
##         rho
## -0.04593898

cor.test(fun4, tax3,method="spearman")##p = 0.03376 rho = 0.6137805

## Warning in cor.test.default(fun4, tax3, method = "spearman"): Cannot compute exact p-value with ties

##
##  Spearman's rank correlation rho
##
## data:  fun4 and tax3
## S = 110.46, p-value = 0.03376
## alternative hypothesis: true rho is not equal to 0
## sample estimates:
##       rho
## 0.6137805

cor.test(fun4, tax4,method="spearman")##p = 0.09179 rho = -0.5079563

## Warning in cor.test.default(fun4, tax4, method = "spearman"): Cannot compute exact p-value with ties

##
##  Spearman's rank correlation rho
##
## data:  fun4 and tax4
```

```
## S = 431.28, p-value = 0.09179
## alternative hypothesis: true rho is not equal to 0
## sample estimates:
##        rho
## -0.5079563
```

```
###Example for visualization
correlation<-cbind(fun1,fun2,fun3,fun4,tax1,tax2,tax4)
correlation<-as.data.frame(correlation)
correlation$site<-c("CI","CI","CI","CLP","CLP","CLP","PI","PI","PI","TPC","TPC","TPC")
p1<-ggplot(correlation,aes(x=fun1,y=tax1,colour=site))+geom_point(size=3)+theme_bw()+
  labs(x="Sulfur metabolism",y="Desulfobulbaceae")+
  theme(axis.text.x = element_text(size = 12,face = "bold"),
        legend.text=element_text(size=12),
        legend.title = element_text(size=12),
        axis.text.y = element_text(size=12),
        axis.title.y = element_text(size=12)
  )
p1
```

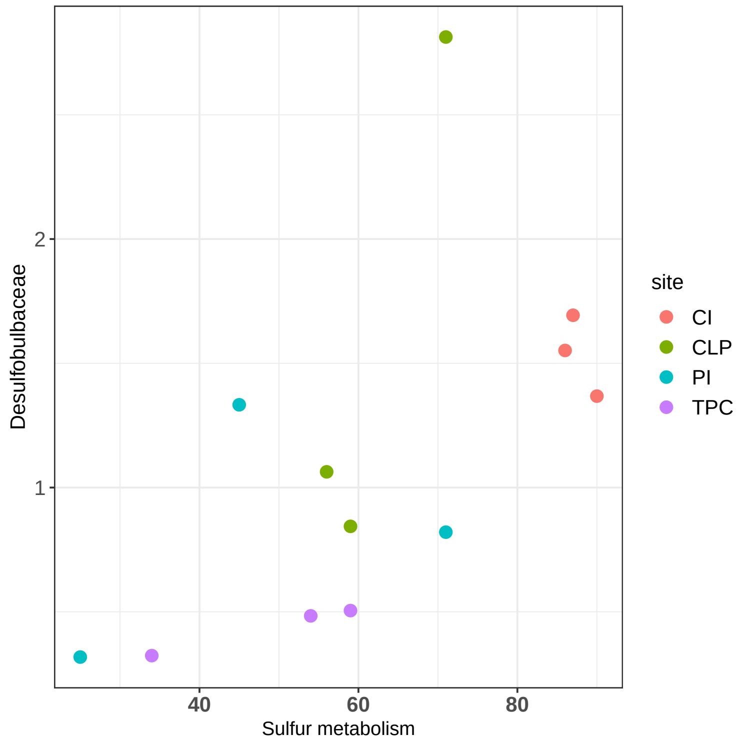

The R session information (including the OS info, R version and all packages used):

```
sessionInfo()
```

```
## R version 3.5.1 (2018-07-02)
## Platform: x86_64-apple-darwin15.6.0 (64-bit)
## Running under: macOS  10.14
##
## Matrix products: default
## BLAS: /System/Library/Frameworks/Accelerate.framework/Versions/A/Frameworks/vecLib.framework/Versions/A/lib
## LAPACK: /Library/Frameworks/R.framework/Versions/3.5/Resources/lib/libRlapack.dylib
```

```
##
## locale:
## [1] en_US.UTF-8/en_US.UTF-8/en_US.UTF-8/C/en_US.UTF-8/en_US.UTF-8
##
## attached base packages:
## [1] grid      stats     graphics  grDevices utils     datasets  methods   base
##
## other attached packages:
##  [1] cowplot_0.9.3   ggpubr_0.1.8    magrittr_1.5    reshape2_1.4.3  dplyr_0.7.6
##  [6] reshape_0.8.7   ggplot2_3.0.0   vegan_2.5-2     lattice_0.20-35 permute_0.9-4
##
## loaded via a namespace (and not attached):
##  [1] tinytex_0.12.1    tidyselect_0.2.4  xfun_0.6          remotes_2.0.4
##  [5] purrr_0.2.5       colorspace_1.3-2  testthat_2.0.0    usethis_1.5.0
##  [9] yaml_2.2.0        mgcv_1.8-24       rlang_0.2.1       pkgbuild_1.0.3
## [13] pillar_1.3.0      glue_1.3.0        withr_2.1.2       sessioninfo_1.1.1
## [17] bindrcpp_0.2.2    bindr_0.1.1       plyr_1.8.4        stringr_1.4.0
## [21] munsell_0.5.0     gtable_0.2.0      devtools_2.0.2    evaluate_0.13
## [25] memoise_1.1.0     labeling_0.3      knitr_1.22        callr_2.0.4
## [29] curl_3.2          parallel_3.5.1    highr_0.7         Rcpp_0.12.18
## [33] scales_0.5.0      backports_1.1.2   desc_1.2.0        pkgload_1.0.2
## [37] fs_1.2.7          digest_0.6.15     stringi_1.2.4     processx_3.1.0
## [41] rprojroot_1.3-2   cli_1.0.0         tools_3.5.1       lazyeval_0.2.1
## [45] tibble_1.4.2      cluster_2.0.7-1   crayon_1.3.4      pkgconfig_2.0.1
## [49] MASS_7.3-50       Matrix_1.2-14     prettyunits_1.0.2 assertthat_0.2.0
## [53] R6_2.2.2          nlme_3.1-137      compiler_3.5.1
```

```
Sys.time()
```

```
## [1] "2019-04-17 11:34:59 HKT"
```
